# Titanium Carbide MXene Synthesis by Etching of Titanium Aluminum Carbide in Acetic Acid Solution

**DOI:** 10.1002/smll.202514731

**Published:** 2026-03-12

**Authors:** Bartosz Gurzęda, Nicolas Boulanger, Gui Li, Mads Ry Vogel Jørgensen, Innokenty Kantor, Igor Baburin, Marta Petre, Marius Enachescu, Alexandr V. Talyzin

**Affiliations:** ^1^ Department of Physics Umeå University Umeå Sweden; ^2^ Institute of Resource Ecology Helmholtz‐Zentrum Dresden‐Rossendorf (HZDR) Dresden Germany; ^3^ MAX IV Laboratory Lund University Lund Sweden; ^4^ Department of Chemistry and iNANO Aarhus University Aarhus C Denmark; ^5^ Ludwig‐Maximilians‐Universität München Sektion Kristallographie München Germany; ^6^ Center for Surface Science and Nanotechnology National University of Science and Technology Politehnica Bucharest Bucharest Romania; ^7^ Academy of Romanian Scientists Bucharest Romania

**Keywords:** environmental friendly, in situ XRD, MXene synthesis, synchrotron, titanium aluminum carbide

## Abstract

Ti‐MXene (T_i3_C_2_T_z_) is the most common member of a larger family of 2D materials widely explored due to a variety of possible applications. MXenes are mostly synthesized using strong acids like HF and HCl or using procedures that require elevated temperatures. Here, we present a new method for Ti_3_C_2_T_z_ preparation with a weak acid solution, which is more beneficial for mass production with reduced environmental impact. It is demonstrated that aluminum can be etched from titanium aluminum carbide (Ti_3_AlC_2_) using ammonium fluoride (NH_4_F) dissolved in an aqueous solution of acetic acid (CH_3_COOH). Optimization of the balance between amounts of water and acetic acid in the etching solution allows for complete etching of Al atoms yielding partially nitrogen terminated MXene in addition to common –O/–OH and –F termination. The mechanism of MXene formation was investigated by the in situ synchrotron radiation X‐ray diffraction (XRD), allowing characterization of “pristine” MXene structure forming directly in the process of Ti_3_AlC_2_ reaction with NH_4_F/CH_3_COOH. In situ XRD analysis also enables identification of the reaction byproducts, thus providing information about the mechanism of MXene formation.

## Graphical abstract

We propose a new method for Ti_3_C_2_F_z_ MXene synthesis using acetic acid as an etching environment. Contrary to the most often used HF/HCl, a weak acid solution is more beneficial for mass production with reduced environmental impact. Optimal balance between the acetic acid and water amounts allows for complete etching of Al atoms yielding partially nitrogen terminated MXene in addition to common –O/–OH and –F termination.

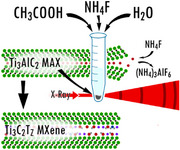

## 1 Introduction

MXenes are a family of 2D materials with the general formula M_n+1_X_n_T_z_, which are prepared by selective etching of the A layer from the as called MAX phases, where M is a transition metal, A stands for an element from 13th or 14th group, and X is C, N, or B atom. T_z_ in the MXene formula is assigned to the various surface termination, such as –O, OH, ─F, or Cl, which are formed during the A‐layer etching [1]. Since the first synthesis of Ti_3_C_2_T_z_ MXene by group of Gogotsi and Barsoum in 2011 [2], the interest in this group of 2D materials has increased enormously with continuous search for new MXene preparation methods and their possible applications. A wide range of uses in energy storage [3, 4], catalysts [5], sensors [6], electromagnetic interface shielding [7], water purification, and so forth was proposed for MXene materials due to their hydrophilicity, 2D morphology, high electrical conductivity, and chemical diversity [1, 8].

Variety of MXene synthesis methods were reported over the past decade, such as chemical etching in aqueous [2, 9, 10, 11, 12] or organic medium [8, 13], electrochemical etching in aqueous [14, 15, 16, 17], organic [18], or molten salt electrolyte [19, 20], etching in molten salts [21, 22], etc. Nevertheless, solution‐based wet‐chemical etching is still most widely explored due to its simplicity and scalability for mass production [23]. It also needs to be noted that the method used for MXene preparation strongly affects its properties, mostly by the different termination of the MXene surface [1, 23].

The earliest MXene synthesis was performed by etching of Ti_3_AlC_2_ in concentrated hydrofluoric acid [2]. During the etching, the surface of MXene is terminated by ─O, ─OH, and –F groups. A new method was later reported to avoid using hazardous HF. In 2014, Ghidiu et al. [9] proposed to etch Ti_3_AlC_2_ by HF formed in situ in a solution of LiF in hydrochloric acid. It was also discovered that this method generates “clay‐like” MXene intercalated by Li, which makes it easier to disperse in water [10]. Some other fluorides, such as NaF [24], FeF_3_ [25], CoF_3_ [26], or HBF_4_ [27, 28] mixed with HCl were also examined for Ti_3_AlC_2_ etching. Halim et al. [29] successfully synthesized Ti_3_C_2_T_z_ using 1 M NH_4_HF_2_. The MXene prepared by this method was characterized by enlarged interlayer spacing due to the intercalation of ammonia and ammonium ions. In 2016, Wang et al. [30] etched the Al layer from MAX phase by hydrothermal treatment of Ti_3_AlC_2_ in NH_4_F solution at 150°C. The synthesized Ti_3_C_2_T_z_ was characterized by an interlayer spacing similar to that of the MXene produced by ammonium bifluoride. The same year Xuan et al. [31] reported the preparation of fluorine‐free titanium carbide nanosheets by the extraction of the Al layer using 25% aqueous solution of tetramethylammonium hydroxide (TMAOH), which is commonly used for Ti_3_C_2_T_z_ delamination [11]. It is worth noting that Ti_3_AlC_2_ was pretreated with 20% HF for a brief period. It should be noted that TMAOH is a strong base, thus suggesting reactions rather different compared acidic etching with HF. Selective etching of Ti_3_AlC_2_ in a mixture of tetramethylammonium fluoride (TMAF) and HCl was also proposed by Kotasthane et al. [32]. The process was performed at room temperature with an MXene yield of about 30 wt% relative to the parent MAX phase. In 2018, Li et al. [33] reported the fluorine‐free synthesis of Ti_3_C_2_ MXene by hydrothermal treatment of MAX phase in 27.5 M NaOH at 270°C. High temperature as well as high sodium hydroxide concentration were crucial for successful Ti_3_C_2_T_z_ formation. Water‐free ionothermal synthesis of Ti_3_C_2_ MXene in deep eutectic solvent (DES) was developed by Wu et al. [34]. Ti_3_AlC_2_ was etched in a mixture of choline chloride, oxalic acid, and NH_4_F at 120°C for 24 h. An enlarged interlayer spacing was achieved due to the intercalation of choline ions. In 2020, Guo et al. [35] used H_2_SO_4_ instead of HCl to form HF in situ in an acid/LiF mixture. The Ti_3_C_2_ MXene prepared using this method exhibited a slightly larger interlayer spacing compared to the LiF+HCl etched one, indicating −SO_4_ MXene surface termination. A new approach for Ti_3_AlC_2_ etching was proposed by Jee et al. [36]. Instead of a strong acid like HF, HCl, or H_2_SO_4_ they proposed to use weaker H_3_PO_4_ as the etching agent and CuF_2_ as the HF source. After etching, Cu was intercalated between Ti_3_C_2_ layers, which were extracted using maleic acid. The produced MXene contained a small portion of ‐PO_4_ termination groups. Shah et al. [37] reported Ti_3_AlC_2_ etching using citric acid (CA) mixed with NH_4_HF_2_ or NH_4_F at room temperature, followed by TMAOH delamination. The Ti_3_C_2_ MXene surface produced via etching with CA and NH_4_HF_2_ was enriched in ─COOH termination. However, Ti_3_AlC_2_ etching using CA and NH_4_F mixture was incomplete and parts of the material characterization (XPS) not valid [37]. Overall, either strong acid or elevated temperatures have so far been used in most of the proposed MXene synthesis methods.

In this work, we propose a new approach to synthesize Ti_3_C_2_T_z_ MXene at ambient temperatures using a more environmentally friendly weak acid. In situ and ex situ experiments demonstrate successful etching of the aluminum layer from Ti_3_AlC_2_ by NH_4_F dissolved in aqueous acetic acid. Moreover, our experiments demonstrate the importance of adding water to the acid in a certain proportion to achieve a higher yield of MXene. This counter‐intuitive result demonstrates that concentrated acetic acid is not suitable for efficient synthesis of MXene while a certain degree of dilution is required for optimization of the MXene synthesis. The MAX etching in CH_3_COOH/H_2_O mixtures with optimal water content allows for full extraction of the Al layer, yielding Ti_3_C_2_T_z_ MXene. Additionally, partial termination of MXene surface by nitrogen was revealed by XPS analysis. The in situ synchrotron radiation X‐ray diffraction (XRD) investigation of the Ti_3_AlC_2_ etching in CH_3_COOH/H_2_O mixture showed that Al reacts with ammonium and fluoride ions, forming mainly ammonium aluminum hexafluoride as a byproduct, which can be easily removed by water washing.

## 2 Results and Discussion

Experiments with the etching of Ti_3_AlC_2_ were performed in this study, first using NH_4_F dissolved in pure acetic acid, and sets of etching tests with water added to the etching solutions in different proportions. As described below, complete conversion of the MAX phase into MXene was achieved after optimizing two parameters: The amount of water added to the NH_4_F/CH_3_COOH and the duration of etching treatment. Bulk samples were synthesized using the proposed procedure and studied after washing with water and drying. An additional set of experiments was also performed using in situ XRD directly during the etching reaction (see Figure S1). The MXene samples were named using the formula MX‐*a*AcA*b*H2O‐*c*d, where *a*:*b* is the CH_3_OOH:H_2_O volume ratio and *c* is the etching duration in days. Detailed information about the etchant composition is given in Table S1.

**FIGURE 1:**
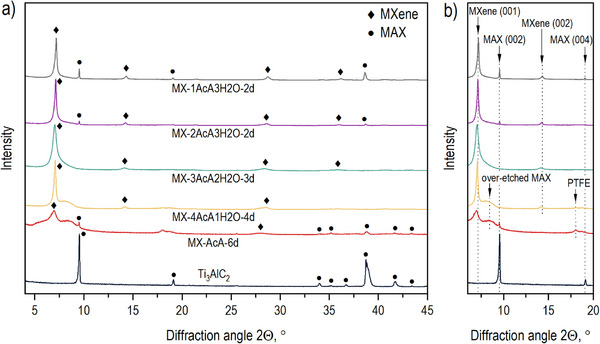
The XRD patterns recorded for the MAX phase after etching by NH_4_F in different acetic acid solutions. (a) shows the region between 4 and 45°, and (b) presents lower angle range of 2Θ.

The influence of water content added to the acetic acid on the etching of the titanium aluminum carbide MAX phase by NH_4_F was investigated using XRD (Figure 1). Analysis of these data recorded from MX‐AcA‐6d sample shows that the (002) and (104) reflections of the MAX phase precursor are still present after 6 days of etching, indicating that full etching of the Al layer from Ti_3_AlC_2_ by NH_4_F in pure CH_3_COOH cannot be achieved even after long‐time treatment. The sample etched in pure acetic acid for a prolonged time also shows the (001) reflection assigned to MXene, at ∼6.9°. This reflection corresponds to a d‐spacing of 12.8 Å, and it is slightly higher than the one reported for the Li‐intercalated Ti_3_C_2_ MXene (12.2–12.4 Å) [9, 38]. Since the method studied here is Li‐free, the experimentally observed d(001) indicates the presence of some cations intercalated between the MXene layers (e.g., ammonium) [29, 30], or suggests functionalization of Ti_3_C_2_ sheets with larger functional groups, other than ─F or –OH, e.g., due to acetylation. There is also an additional broad feature centered approximately at 8.4°, which is most likely caused by over‐etching of the MXene surface as it emerges only after prolonged exposure of the sample to the etching solution. It should be noted that samples stirred for even longer times (up to 6 days) also showed additional reflections from the polytetrafluoroethylene (PTFE) contamination due to long‐time continuous magnetic stirring of the solution in the PTFE container. It can be concluded that etching of Ti_3_AlC_2_ in pure acetic acid using ammonium fluoride is not possible in the tested conditions.

**FIGURE 2:**
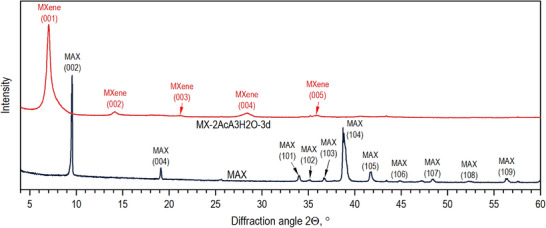
XRD pattern of MAX phase (black curve) and MAX etched by NH_4_F in 60% aqueous acetic acid by volume for 3 days (red curve).

The XRD pattern for the sample etched in 80% aqueous acetic acid (by volume) shows that the presence of water accelerates the etching of Al from the MAX phase, allowing for its complete extraction in 4 days. Notably, some contamination with PTFE was also observed, however the signal from PTFE is weaker as compared to the MXene sample etched for 6 days. Further increase of water content in acetic acid to 40% by volume allows for fully etching the aluminum in a shorter time of 3 days (Figure 2). In this case, contamination with PTFE was avoided. The asymmetric shape of the signal originating from MXene (001) reflections suggests some insignificant over‐etching, introducing disorder into the MXene structure. The c‐unit cell parameter calculated using (00l) set of reflections for MX‐3AcA‐2H2O‐3d is equal to 12.6 Å, the same as in the sample etched in pure acetic acid. The MXene (001) reflection for the Ti_3_AlC_2_ etched for 2 days in 60% and 80% aqueous CH_3_COOH solutions has a more symmetric shape, indicating that over‐etching is avoided. However, the (001) reflection from Ti_3_AlC_2_ is still present in both samples. Moreover, when the etching was extended to 3 days (Figures S2 and S3), XRD (001) and (104) reflections originating from Ti_3_AlC_2_ can still be observed.

**FIGURE 3:**
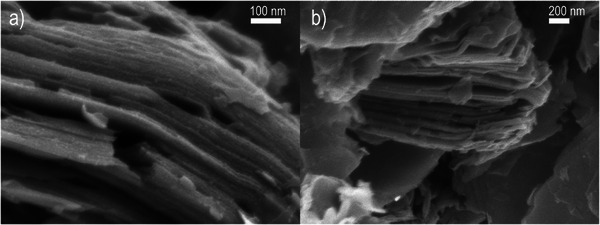
Characterization of sample etched for 3 days in 40% aqueous acetic acid using electron microscopy: SEM images (a and b).

**FIGURE 4:**
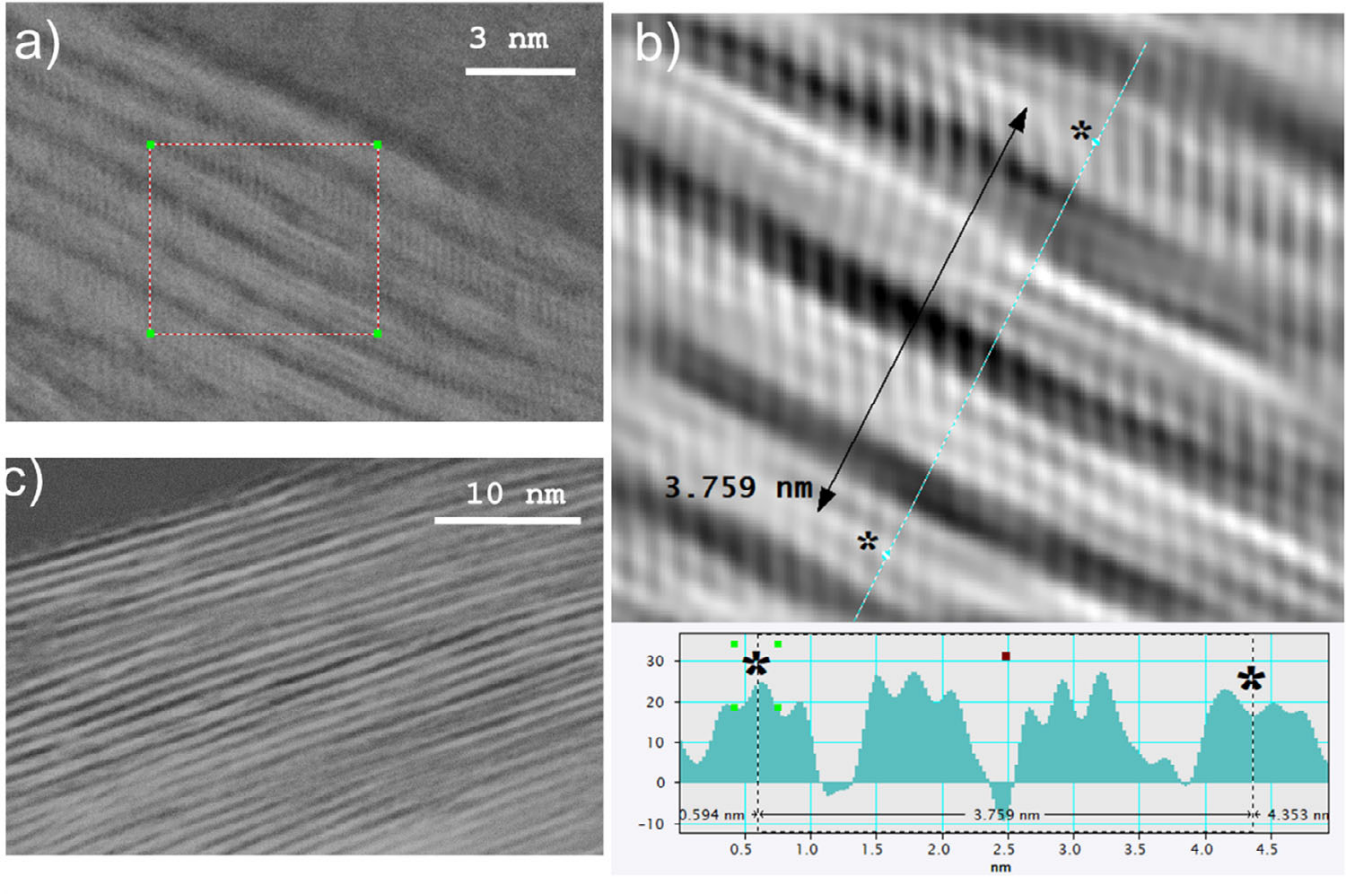
Ultra high resolution STEM images: (a) image recorded with x8000K magnification, (b) magnified area shown in the image (a) profile showing Ti_3_C_2_ layers separated by interlayer spaces and contrast profile showing c‐lattice of 12.53 Å in a good agreement with XRD data (12.6 Å); (c) image recorded using x3000K magnification showing c‐lattice of MXene sample.

Additional characterization of the sample etched for 3 days was performed using electron microscopy (Figures 3 and 4). Images collected using SEM revealed typical for MXene layered morphology (Figure 3) originating from the etching treatment. Ultra‐high resolution STEM images also confirmed successful MXene synthesis (Figure 4).

Ultra high resolution images (Figure 4) clearly show a layered structure along c‐lattice of MXene structure composed by Ti_3_C_2_ layers and the absence of Al between these layers. The area shown in Figure 4a is magnified in Figure 4b with a contrast profile that allows to evaluate c‐unit cell parameter. The contrast profile shown in Figure 4b includes four Ti_3_C_2_ layers and three interlayers shows 37.6 Å width, thus corresponding to c‐unit cell parameter 12.53 Å. This value is in good agreement with d(001) = 12.6 Å value recorded from this sample using XRD.

The etching of Ti_3_AlC_2_ MAX phase in different CH_3_COOH/H_2_O solutions shows that water promotes the extraction of Al. The etching of the MAX phase with an NH_4_F solution in pure acetic acid is too slow and incomplete, even after several days. These data suggest that water facilitates penetration of in situ formed HF into the inner spaces of MAX phase interlayers. However, the exact mechanism of water content influence on the etching reaction and formation of MXene could not be resolved using ex situ experiments. On the other hand, when the concentration of acetic acid is too low, the acidic medium is not strong enough to fully etch the Al layer from the inner spaces of MAX phase.

**FIGURE 5:**
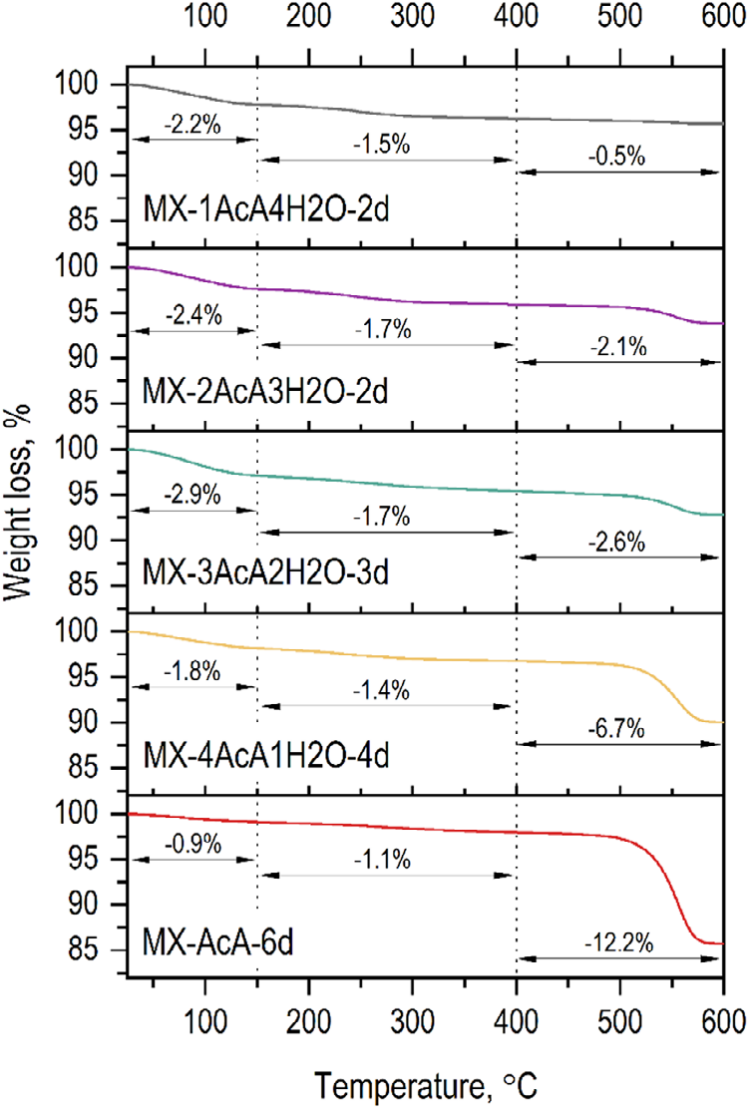
TG curves recorded for the MXenes prepared after etching by NH_4_F in different acetic acid solutions.

Thermogravimetric (TG) analysis of the MXenes prepared in different acetic acid solutions shows three temperature ranges of weight loss during thermal treatment of the samples (Figure 5). The first one, when the MXene is heated to 150°C, is assigned to water release from the Ti_3_C_2_T_z_ surface. It can be noted that the MXene prepared using increased water content in the etching solution is more hydrophilic, as evidenced by the sorption of a larger amount of water from air at ambient humidity. This result suggests that higher water concentration in the solution leads to a higher concentration of oxygen surface termination [11]. The second loss in mass, of ∼1.5%, observed for all the MXenes between 150°C and 400°C, is attributed to the thermal decomposition of less stable oxygen functional groups [39, 40]. The last mass decrease between 400°C and 600°C is likely to be caused by thermal decomposition of fluorine functionalization groups (including also some PTFE contamination in samples with very long etching time). Based on the literature on PTFE thermal decomposition starts at ∼550°C [41] hence TG analysis is in agreement with XRD data. The removal of acetyl groups is also likely to occur in this temperature region (200°C–400°C) [42]. It should be noted that decomposition of acetyl groups and organic modifiers on metal surfaces is typically found in 200°C–600°C interval.

The MXene prepared by etching for 3 days using NH_4_F dissolved in 60% CH_3_OOH is characterized by the full extraction of aluminum and the highest purity of the MXene. Therefore, it was chosen for more detailed characterization.

**FIGURE 6:**
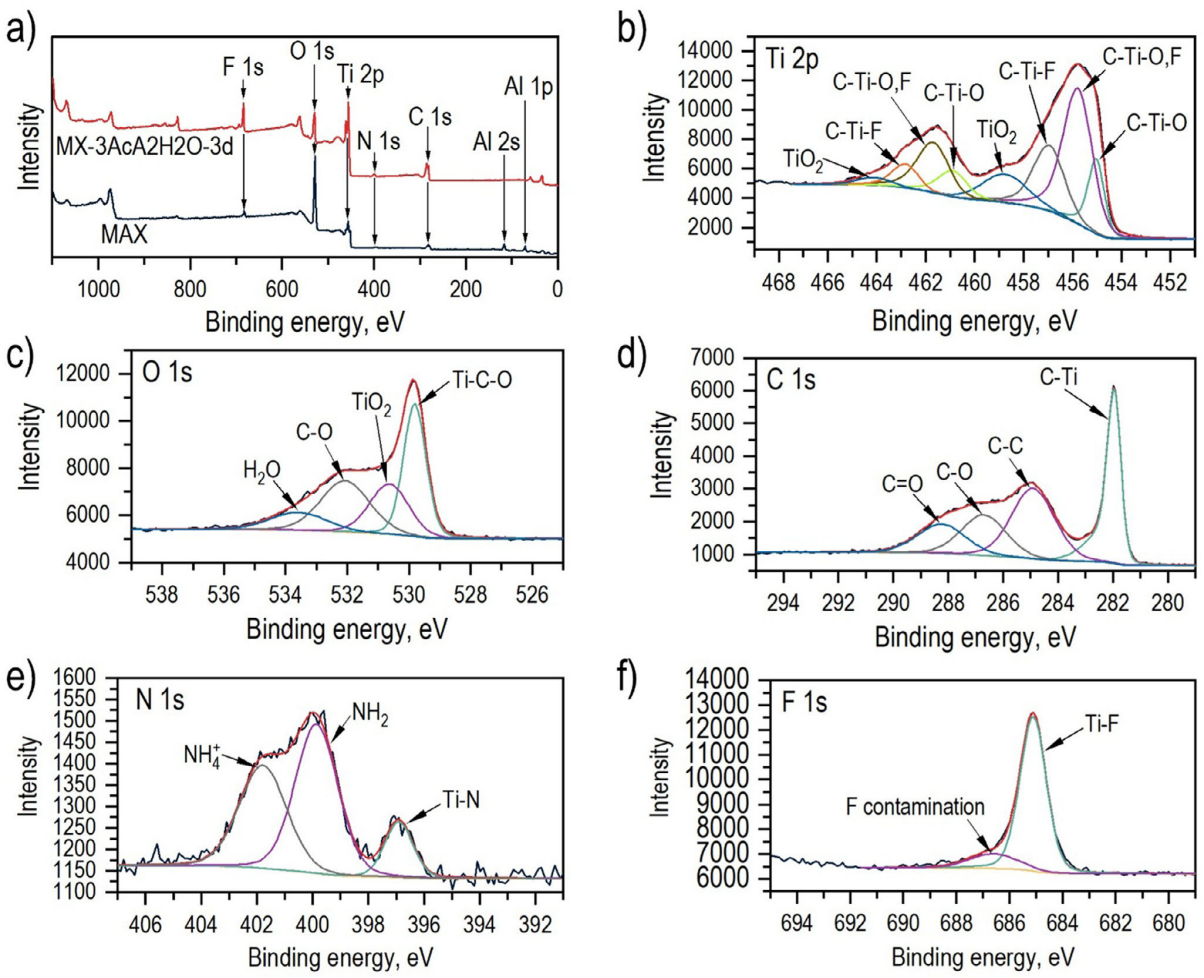
XPS analysis of the MXene synthesized by NH_4_F etching in 60% acetic acid. (a) shows the survey spectra of MAX phase and MXene. High‐resolution spectra of (b) Ti 2p, (c) O 1s, (d) C 1s, (e) N 1s, and (f) F 1s regions recorded for MX‐3AcA2H2O‐3d.

The XPS analysis of Ti_3_C_2_ MXene prepared by 3 days treatment using a NH_4_F solution in 60% acetic acid (Figure 6) shows that Al was almost completely etched from the MAX phase (Figure S4). The XPS survey spectrum of Ti_3_C_2_T_z_ (Figure 6a) indicates the surface termination of MXene by fluorine, oxygen, and nitrogen. The high‐resolution spectrum of the Ti 2p region exhibits typical chemical states of titanium (Figure 6b). The deconvoluted Ti 2p signal is composed of four sets of doublets, which correspond to Ti─O, Ti─O,F, Ti─F, and TiO_2_ at 455.0 eV (460.9 eV), 455.8 eV (461.7 eV), 457.0 eV (462.9 eV), and 458.8 eV (464.1 eV), respectively [43, 44, 45, 46]. The presence of the doublet assigned to TiO_2_ indicates that the MXene surface is partially oxidized to titanium dioxide (The XPS data show that about 10% of the surface titanium was oxidized to TiO_2_). A signal corresponding to Ti─N bond is observed in the N 1s spectrum at 396.9 eV (Figure 6e) [47, 48], which indicates partial MXene surface termination by nitrogen. Two additional signals at 399.9 and 401.8 eV are assigned to amine groups and ammonium, respectively. This result is in agreement with the XRD analysis, revealing an enlarged interlayer distance between MXene layers caused by the presence of NH_3_ and NH_4_^+^ groups [29, 30]. The high‐resolution C 1s spectrum shows a clear signal corresponding to C─Ti bonds at 282.0 eV (Figure 6d). Further deconvoluted signals at 284.9 eV originate from C─C bonds and two next deconvoluted signals, at 286.7 and 288.2 eV are assigned to the oxygen‐bonded carbon [43, 45, 49]. The F 1s region comprises two signals at 685.1 and 686.6 eV associated with Ti─F and F contamination, respectively (Figure 6f). The presence of signal at 686.2 eV on F 1s spectrum is most likely originating from residual contamination, like AlF_3_ and TiO_2‐x_F_2x_ [45]. The deconvoluted O 1s region consists of four peaks. The signal at 529.8 eV is assigned to Ti─C─O. The next deconvoluted signal at 530.7 eV arises from TiO_2_. The last two signals at 532.1 and 533.6 eV are assigned to C─O bonds and adsorbed H_2_O on MXene surface, respectively. The total atomic surface composition of MX‐3AcA2H2O‐3d (Table 1) shows that the MXene prepared in our experiment is mostly terminated by oxygen. Fluorine termination reaches 8 atomic%. The XPS results reveal that around 3 atomic% of nitrogen is present on the prepared Ti_3_C_2_T_z_ surface. It can be noted that high atomic carbon content suggests partial termination of prepared MXene surface by COOH groups.

**TABLE 1:** XPS fitting results for MX‐3AcA2H2O‐3d sample.

Element:	Ti	C	O	F	N
Overall atomic%:	19.22	51.26	18.82	7.87	2.83

**FIGURE 7:**
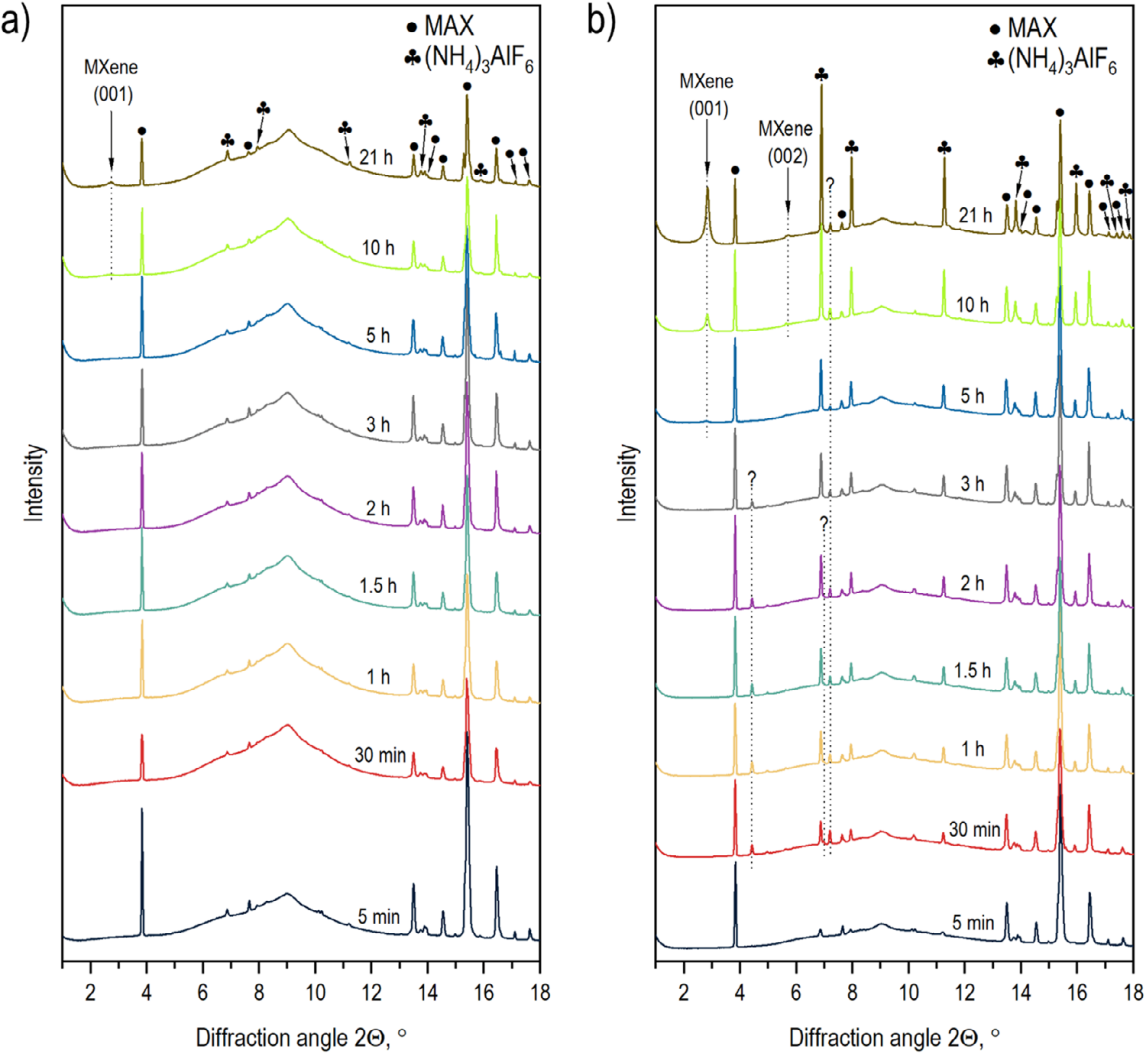
In situ synchrotron radiation XRD study of Ti_3_AlC_2_ etching by ammonium fluoride in (a) pure acetic acid and (b) in 60% acetic acid (λ = 0.61992 Å).

As explained above, using only ex situ characterization of the samples produced by etching the MAX phase with NH_4_F solutions in acetic acid, we were unable to evaluate the reaction mechanism and, in particular, the effect of added water on the etching reaction rate. However, the in situ XRD study of the etching reaction showed that water likely assists in the dissolution of reaction by‐products, thus providing easier access for HF into the interlayer space. In situ synchrotron radiation XRD enables the direct evaluation of MXene structure during the etching reaction, prior to water washing, and in a solution‐immersed state. The analysis of XRD patterns recorded during MAX phase etching with NH_4_F mixed with pure acetic acid (Figure 7a) revealed new reflections identified as ammonium aluminum hexafluoride ((NH4)_3_AlF_6_) (Figure S5). Therefore, in situ experiments reveal that the reaction between in situ formed HF, NH_4_^+^, and aluminum results in the formation of a solid byproduct insoluble in pure acetic acid. The (001) reflection of MXene was first observed after ∼10 h of etching, but even after 21 h, the (001) MXene reflection remained relatively weak. This result is in agreement with the analysis of ex situ XRD data, confirming the slow kinetics of the Al etching by NH_4_F in pure acetic acid. However, in situ data allow us to explain the slow etching by the presence of solid (NH4)_3_AlF_6_, which is likely to block access of HF into the Ti_3_C_2_ interlayers after the removal of Al.

The etching of the Al layer from the MAX phase by NH_4_F in 60% acetic acid was significantly faster compared to the reaction performed in pure acetic acid (Figure 7b). The (001) MXene reflection was observed as early as ∼5 h of etching, and after 21 h, a significant portion of MAX had transformed into Ti_3_C_2_T_z_. As in the case of pure acetic acid, the XRD reflections assigned to (NH4)_3_AlF_6_ can also be observed in 60% aqueous solution from the beginning of etching. Moreover, the relative intensity of these reflections is significantly higher, reflecting the removal of larger amounts of Al from the MAX phase structure and a higher yield of MXene. Relatively sharp reflections of (NH4)_3_AlF_6_ indicate that crystallization of this byproduct occurs outside of narrow MXene interlayers, forming bulk powder precipitates in the inter‐crystallite space. It is logical to suggest that water added to acetic acid facilitates the dissolution of (NH4)_3_AlF_6_ and its removal from the interlayer space of newly formed MXene. In particular, it could also be anticipated that swelling in water further expands the MXene interlayers, making access for the HF acid even easier. Surprisingly, the interlayer distance of “pristine” Ti_3_C_2_T_z_ (immersed in etching solution) was found to be the same in solutions prepared using pure acetic acid and acetic acid with added water (∼12.9 Å). Moreover, one additional solid byproduct was identified using in situ XRD data recorded in an experiment with water‐added solutions. Additional new reflections are observed at 4.4°, 5.0°, 7.0°, and 7.2° (8.03, 7.15, 5.08, and 4.93 Å, respectively) as early as ∼10 min of etching. After 3 h, the first two reflections disappeared, while the next two remained present after 21 h of etching (Figure S6). These two signals at 7.0° and 7.2° are originating from ammonium aluminum pentafluoride hydrate ((NH_4_)_2_AlF_5_·H_2_O), and its formation is due to the presence of water in acetic acid (Figure S5). We were not able to assign the first two reflections at 4.5° and 5.0° to any specific crystalline structure, but it is most likely that those reflections originate from hydrated salts that form during the etching of aluminum in the presence of water, such as aluminum acetate hydrate or aluminum fluoride hydrate. We suspect that the formation of additional byproducts facilitates easier access of the in situ formed HF to the interlayer spaces of the MAX phase, thereby accelerating Al extraction. All byproducts formed during the MAX etching process using NH_4_F in an acetic acid solution are soluble in water and can be easily removed after the etching completion (Figure S6).

Similar to the etching with pure acetic acid, the d(001) value of MXene, recorded directly during etching in a 60% aqueous solution, is approximately 12.9 Å. This value is also very close to the d(001) recorded from MXene washed with water (after drying). Therefore, the post‐synthesis treatment of MXene produced by NH_4_F/CH_3_COOH with water is important only for the removal of reaction byproducts and does not further modify the structure of MXene.

The interlayer distance of MXene prepared by NH_4_F/CH_3_COOH (∼12.9 Å) is similar to the d(001) of MXene produced by LiF+HF etching (∼12.5 Å), which is, in the latter case, believed to be intercalated by Li. The smallest interlayer distance provided by the d(001) value is found for MXene synthesized in HF. In this case, the MXene layers are mostly terminated by fluorine, and the interlayers are free from intercalated species (∼10.2 Å) [50]. Therefore, the XRD data suggest that the MXene synthesized in our study has an interlayer distance expanded by ∼2.6 Å compared to non‐intercalated ─F and ─OH‐terminated MXene [2, 51]. This expansion could be explained either by intercalation or by functionalization with larger functional groups. For example, intercalation with ammonia (2.6 Å in diameter) provides a close match and can be supported by the presence of nitrogen detected by XPS. An alternative explanation could be the expansion of the MXene interlayer width due to the functionalization of Ti_3_C_2_ sheets with acetyl groups replacing the hydroxyl groups. Our earlier experiments with the acetylation of graphene oxide demonstrated an increase in interlayer distance by ∼2.2 Å, which remained stable even after the sample was immersed in water [52]. The partial acetylation of MXene is in agreement with XPS data, which show an excess of carbon relative to the expected Ti_3_C_2_ stoichiometry.

**FIGURE 8:**
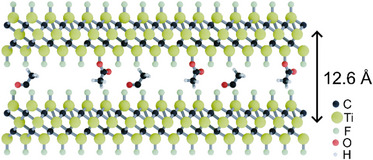
Schematic structural model of acetylated MXene.

Simplified structural model of acetylated MXene is shown in Figure 8. For simplicity, the model shows only ─F termination of Ti_3_C_2_ sheets. The increase of interlayer spacing by 2 Å is achieved by replacement of both Ti─F and Ti─O bonds (same length of ∼2 Å) with Ti─O─C(O)CH_3_. The model is constructed using one acetyl group per twenty Ti atoms, which is in agreement with the amount of carbon found in our samples by XPS. The model is over‐simplified because acetyl groups are expected to be completely disordered, similarly to the absence of ordering for ─F, ─O─, and ─OH termination groups in a standard MXene structure.

It is likely that the density of acetyl groups terminating 2D sheets can be varied depending on the conditions of synthesis. It is also possible that both intercalation of ammonium and acetylation of Ti_3_C_2_ sheets occur simultaneously.

The overall transformation of the MAX phase into MXene can be suggested to involve several reactions, as discussed below. Based on our in situ XRD data, one of the main products of the etching reaction is (NH_4_)_3_AlF_6_. Therefore, the sequence of reactions can be proposed as follows. For pure acetic acid, Al is first etched by in situ formed HF:

(1) $$CH_{3}\mathrm{COOH}+NH_{4}F\to CH_{3}\mathrm{COO}\;NH_{4}F+\mathrm{HF}$$

(2) $$Ti_{3}\mathrm{Al}C_{2}+4\mathrm{HF}\to Ti_{3}C_{2}F+\mathrm{Al}F_{3}+2H_{2}$$

Aluminum fluoride reacts further with ammonium fluoride, forming (NH_4_)_3_AlF_6_:

(3) $$\mathrm{Al}F_{3}+3NH_{4}F\to {\left(NH_{4}\right)}_{3}\mathrm{Al}F_{6}$$

Equation (1) suggests the presence of acetyl anions and ammonia cations, which are likely to react with Ti_3_C_2,_ resulting in partial acetylation or amination in addition to fluorine termination:

(4) $$\begin{array}{cc} Ti_{3}\mathrm{Al}C_{2} & +2CH_{3}\mathrm{COOH}+3NH_{4}F\to Ti_{3}C_{2}NH_{2}+\mathrm{Al}F_{3}\\ & +2CH_{3}\mathrm{COON}H_{4}+2H_{2} \end{array}$$

(5) $$\begin{array}{cc} Ti_{3}\mathrm{Al}C_{2} & +2CH_{3}\mathrm{COOH}+NH_{4}F+2\mathrm{HF}\to Ti_{3}C_{2}\mathrm{OOC}H_{3}\\ & +\mathrm{Al}F_{3}+CH_{3}\mathrm{COON}H_{4}+2H_{2} \end{array}$$

When the water solution, instead of pure acetic acid, is used, the additional byproduct identified as (NH_4_)_2_AlF_5_·H_2_O forms:

(6) $$\mathrm{Al}F_{3}+2NH_{4}F+H_{2}O\to {\left(NH_{4}\right)}_{2}\mathrm{Al}F_{5}\cdot H_{2}O$$

The formation of other hydrates, such as aluminum fluoride hydrate, should also be taken into consideration:

(7) $$\mathrm{Al}F_{3}+xH_{2}O\to \mathrm{Al}F_{3}\cdot xH_{2}O$$

Equations (6) and (7) suggest that water is an important addition to acetic acid, as it allows for the formation of hydrated byproducts and dissolves them, which would otherwise likely block entrance into MXene interlayers.

It is important to note that the reaction of HF with Al of the MAX phase occurs within a relatively narrow space of MXene inter‐layers formed in the process of etching. The width of MXene interlayers, estimated using our XRD data, is approximately 2.2 Å in the solution‐immersed state, which is barely sufficient for the penetration of HF in a single layer of solution. The narrow width of interlayers provides limitations for the size of molecules capable of diffusing in and out of the c‐lattice. For example, the size of (NH_4_)_3_AlF_6_ is relatively large (∼5.4 Å), thus exceeding the width of MXene interlayers. Therefore, reaction described in the Equation (1) is likely to occur outside of MXene interlayers when AlF_3_ is extracted from the lattice. Moreover, the size of ammonia cations is also larger than the width of MXene interlayers even in the completely dehydrated state (∼3.5 Å).

Therefore, we suggest that only HF penetrates the interlayer space of Ti_3_AlC_2_ during the Al extraction process, possibly also without the use of water. Th ereaction step describe din equation (2) occurs inside a confined space formed by Al extraction, while reactions given in  equations (3) and (6) are likely to occur outside of the MXene interlayers when AlF_3_ diffuses out of the lattice. This idea is supported by XRD data showing relatively sharp and strong reflections (NH_4_)_3_AlF_6,_ proving that it forms as a bulk powder.

**FIGURE 9:**
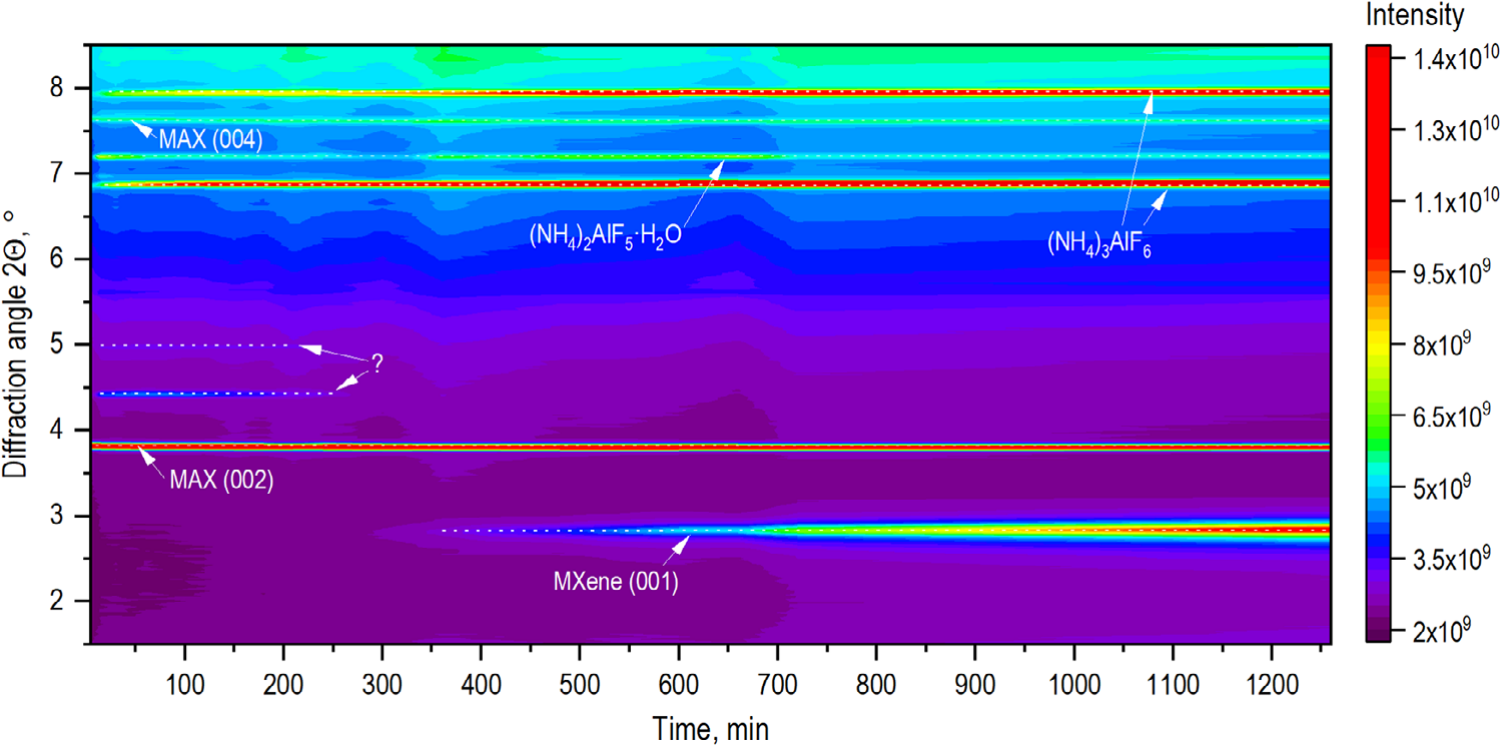
Time‐resolved map of XRD patterns recorded during MAX phase etching by NH_4_F in in 60% acetic acid (λ = 0.61992 Å).

It is also remarkable that the massive formation of (NH_4_)_3_AlF_6_ is evident almost immediately after the start of the etching reaction and long before the first reflections of MXene appear about 6 h later (Figure 9). This observation implies that some non‐crystalline phases must be present in the studied sample. This could be either an amorphous phase of MXene (e.g., completely delaminated) or some extremely disordered phase formed by the extraction of Al from the Ti_3_AlC_2_ lattice, but not yet converted into the MXene. It should be emphasized that the structure of precursor MAX phase and MXene remained unchanged during the whole duration of the experiment, as it follows from negligibly small shifts in positions of XRD reflections from these phases. Therefore, the formation of MXene in several steps, e.g., as first only ─F terminated and then acetylated at later stages of the reaction, can be ruled out. We note that synchrotron XRD is a powerful technique that typically allows for the detection of even some smaller amounts of crystalline impurity phases.

Finally, the in situ XRD data actually demonstrate the presence of some additional phase at the early stages of the etching reaction. The main reflections of this phase (8.03 and 7.15 Å) were found during the first 3 h of the reaction (see the heat map in Figure 9) and disappeared simultaneously with a strong increase in intensity of the (NH_4_)_3_AlF_6_ reflections before the formation of the MXene structure. We considered several possible alternatives (e.g., AlF_3_, NH_4_F), but so far we have not been able to identify the origin of these reflections. It can be noted that the d‐spacing of 8.03 Å is only slightly smaller than the d(002) value of precursor MAX‐phase (∼9.32 Å) and might originate from some intermediate phase formed after removal of Al but not yet formed MXene. Therefore, the formation of crystalline MXene by Equation (2) possibly involves some additional, yet unidentified, intermediate steps.

## 3 Conclusions

We demonstrate a novel method for synthesizing Ti_3_C_2_T_z_ MXene using NH_4_F dissolved in aqueous acetic acid. The proposed method, based on a weak acid, provides a more environmentally friendly route to etch aluminum from Ti_3_AlC_2_ MAX phase as an alternative to commonly used strong acids such as HF and HCl. The etching of the MAX phase using an NH_4_F solution in 60% acetic acid results in the full extraction of the Al layer, yielding Ti_3_C_2_T_z_ MXene with an interlayer spacing of 12.6 Å. The MXene sheets synthesized by our method are terminated mainly by oxygen‐ and fluorine‐containing groups. However, partial acetylation and ammonium termination are also likely, considering the expansion of c‐lattice by ∼2 Å relative to fluorine‐terminated MXene. We demonstrate that etching of Ti_3_AlC_2_ by NH_4_F in pure (water‐free) acetic acid is significantly slower and incomplete, suggesting that the accumulation of reaction byproducts blocks the entrance of the etching solution into the interlayers. On the other hand, when the acetic acid concentration is too low, Al cannot be fully extracted. Therefore, an optimal balance between the acetic acid and water amounts is needed to fully transform MAX into MXene. Our data suggest that water helps to dissolve Al‐containing reaction byproducts and to remove them from interlayers, thus providing easier access of the etching agent to deeper parts of non‐etched Ti_3_AlC_2_.

In situ synchrotron radiation XRD data recorded in the process of MAX phase etching with NH_4_F/CH_3_COOH mixture reveal a surprisingly long delay between almost immediate (within minutes) appearance of Al‐containing reaction products and first indications of MXene formation only after about 6 h. It is found that Al removed from the MAX phase by etching is precipitated as a solid (NH_4_)_3_AlF_6_ and (NH_4_)_2_AlF_5_·H_2_O_._ The clear evidence of Al removal from the MAX phase, in combination with the absence of MXene formation for several hours, suggests the existence of intermediate phases involved in the reaction. However, the structures of MXene and MAX phase remain unchanged during the whole etching process, indicating that the intermediate structures formed by the removal of Al from the MAX phase must be non‐crystalline.

Limitations provided by the rather narrow width of MXene interlayers in the solution‐immersed state suggest that only the main etching reaction occurs inside the space confined between Ti_3_C_2_ sheets. In contrast, formation of (NH_4_)_3_AlF_6_ and (NH_4_)_2_AlF_5_·H_2_O occurs in a bulk state outside of the interlayers. It can be concluded that in situ XRD monitoring of MAX phase etching reactions provides valuable insight into the mechanism of MAX phase etching and MXene formation [53, 54].

## Conflicts of Interest

The authors declare no conflicts of interest.

## Supporting information




**Supporting File**: smll73078‐sup‐0001‐SuppMat.docx.

## Data Availability

The data that support the findings of this study are available from the corresponding author upon reasonable request.

## References

[1] A. VahidMohammadi , J. Rosen , and Y. Gogotsi , “The World of Two‐dimensional Carbides and Nitrides (MXenes),” Science 372 (2021): abf1581. 10.1126/science.abf1581 34112665

[2] M. Naguib , M. Kurtoglu , V. Presser , et al., “Two‐Dimensional Nanocrystals Produced by Exfoliation of Ti_3_AlC_2_ ,” Advanced Materials 23 (2011): 4248–4253. 21861270 10.1002/adma.201102306

[3] X. Li , Z. Huang , C. E. Shuck , G. Liang , Y. Gogotsi , and C. Zhi , “MXene Chemistry, Electrochemistry and Energy Storage Applications,” Nature Reviews Chemistry 6 (2022): 389–404. 37117426 10.1038/s41570-022-00384-8

[4] Y. Li , X. Feng , W. Y. Lieu , et al., “MXene‐Based Anode‐Free Magnesium Metal Battery,” Advanced Functional Materials 33 (2023): 2303067.

[5] Y. Zhou , L. Liang , C. Wang , et al., “Precious‐Metal‐Free Mo‐MXene Catalyst Enabling Facile Ammonia Synthesis via Dual Sites Bridged by H‐Spillover,” Journal of the American Chemical Society 146 (2024): 23054–23066. 39133788 10.1021/jacs.4c03998 PMC11345764

[6] S. J. Kim , H.‐J. Koh , C. E. Ren , et al., “Metallic Ti_3_C_2_T_x_ MXene Gas Sensors with Ultrahigh Signal‐to‐Noise Ratio,” ACS Nano 12 (2018): 986–993. 29368519 10.1021/acsnano.7b07460

[7] R. Verma , P. Thakur , A. Chauhan , R. Jasrotia , and A. Thakur , “A Review on MXene and Its″ composites for Electromagnetic Interference (EMI) Shielding Applications,” Carbon 208 (2023): 170–190.

[8] V. Natu , R. Pai , M. Sokol , M. Carey , V. Kalra , and M. W. Barsoum , “2D Ti_3_C_2_T_z_ MXene Synthesized by Water‐free Etching of Ti_3_AlC_2_ in Polar Organic Solvents,” Chem 6 (2020): 616–630.

[9] M. Ghidiu , M. R. Lukatskaya , M.‐Q. Zhao , Y. Gogotsi , and M. W. Barsoum , “Conductive Two‐dimensional Titanium Carbide ‘Clay’ with High Volumetric Capacitance,” Nature 516 (2014): 78–81. 25470044 10.1038/nature13970

[10] M. Ghidiu , J. Halim , S. Kota , D. Bish , Y. Gogotsi , and M. W. Barsoum , “Ion‐Exchange and Cation Solvation Reactions in Ti_3_C_2_ MXene,” Chemistry of Materials 28 (2016): 3507–3514.

[11] M. Alhabeb , K. Maleski , B. Anasori , et al., “Guidelines for Synthesis and Processing of Two‐Dimensional Titanium Carbide (Ti_3_C_2_T_x_ MXene),” Chemistry of Materials 29 (2017): 7633–7644.

[12] N. Xue , X. Li , L. Han , et al., “Fluorine‐free Synthesis of Ambient‐stable Delaminated Ti_2_CT_x_ (MXene),” Journal of Materials Chemistry A 10 (2022): 7960–7967.

[13] H. Shi , P. Zhang , Z. Liu , et al., “Ambient‐Stable Two‐Dimensional Titanium Carbide (MXene) Enabled by Iodine Etching,” Angewandte Chemie International Edition 60 (2021): 8689–8693. 33484049 10.1002/anie.202015627 PMC8048443

[14] S. Yang , P. Zhang , F. Wang , et al., “Fluoride‐Free Synthesis of Two‐Dimensional Titanium Carbide (MXene) Using a Binary Aqueous System,” Angewandte Chemie International Edition 57 (2018): 15491–15495. 30289581 10.1002/anie.201809662

[15] S.‐Y. Pang , Y.‐T. Wong , S. Yuan , et al., “Universal Strategy for HF‐Free Facile and Rapid Synthesis of Two‐dimensional MXenes as Multifunctional Energy Materials,” Journal of the American Chemical Society 141 (2019): 9610–9616. 31117483 10.1021/jacs.9b02578

[16] K. C. Chan , X. Guan , T. Zhang , et al., “The Fabrication of Ti_3_C_2_ and Ti_3_CN MXenes by Electrochemical Etching,” Journal of Materials Chemistry A 12 (2024): 25165–25175.

[17] M. Ostermann , M. Piljević , E. Akbari , et al., “Pulsed Electrochemical Exfoliation for an HF‐Free Sustainable MXene Synthesis,” Small 21 (2025): 2500807. 40159789 10.1002/smll.202500807 PMC12138861

[18] T. Yin , Y. Li , R. Wang , et al., “Synthesis of Ti_3_C_2_F_x_ MXene with Controllable Fluorination by Electrochemical Etching for Lithium‐ion Batteries Applications,” Ceramics International 47 (2021): 28642–28649.

[19] L. Liu , H. Zschiesche , M. Antonietti , et al., “In Situ Synthesis of MXene with Tunable Morphology by Electrochemical Etching of MAX Phase Prepared in Molten Salt,” Advanced Energy Materials 13 (2023): 2203805.

[20] F. Tian , Z. Pang , X. Yu , et al., “Polyanion Electrointercalation Enables Termination‐Tailored Oligolayer Ti_3_C_2_T_x_ MXene Synthesis in Alkali Chloroaluminate Melt,” Angewandte Chemie International Edition 137 (2025): 202514594.. 10.1002/anie.202514594 40944602

[21] L. Liu , M. Orbay , S. Luo , et al., “Exfoliation and Delamination of Ti_3_C_2_T_x_ MXene Prepared via Molten Salt Etching Route,” ACS Nano 16 (2022): 111–118. 34787390 10.1021/acsnano.1c08498

[22] M. Li , J. Lu , K. Luo , et al., “Element Replacement Approach by Reaction with Lewis Acidic Molten Salts to Synthesize Nanolaminated MAX Phases and MXenes,” Journal of the American Chemical Society 141 (2019): 4730–4737. 30821963 10.1021/jacs.9b00574

[23] S. Jin , Y. Guo , F. Wang , and A. Zhou , “The Synthesis of MXenes,” MRS Bulletin 48 (2023): 245–252.

[24] F. Liu , A. Zhou , J. Chen , et al., “Preparation of Ti_3_C_2_ and Ti_2_C MXenes by Fluoride Salts Etching and Methane Adsorptive Properties,” Applied Surface Science 416 (2017): 781–789.

[25] X. Wang , C. Garnero , G. Rochard , et al., “etching Environment (FeF_3_/HCl) for the Synthesis of Two‐dimensional Titanium Carbide MXenes: a Route towards Selective Reactivity vs. water,” Journal of Materials Chemistry A 5 (2017): 22012–22023.

[26] C. B. Cockreham , X. Zhang , H. Li , et al., “Inhibition of AlF_3_·3H_2_O Impurity Formation in Ti_3_C_2_T_x_ MXene Synthesis under a Unique CoF_x_/HCl Etching Environment,” ACS Applied Energy Materials 2 (2019): 8145–8152.

[27] A. Gentile , S. Marchionna , M. Balordi , et al., “Critical Analysis of MXene Production with in‐Situ HF Forming Agents for Sustainable Manufacturing,” ChemElectroChem 9 (2022): 202200891.

[28] C. Peng , P. Wei , X. Chen , et al., “A Hydrothermal Etching Route to Synthesis of 2D MXene (Ti_3_C_2_, Nb_2_C): Enhanced Exfoliation and Improved Adsorption Performance,” Ceramics International 44 (2018): 18886–18893.

[29] J. Halim , M. R. Lukatskaya , K. M. Cook , et al., “Transparent Conductive Two‐Dimensional Titanium Carbide Epitaxial Thin Films,” Chemistry of Materials 26 (2014): 2374–2381. 24741204 10.1021/cm500641a PMC3982936

[30] L. Wang , H. Zhang , B. Wang , et al., “Synthesis and Electrochemical Performance of Ti_3_C_2_T_x_ with Hydrothermal Process,” Electronic Materials Letters 12 (2016): 702–710.

[31] J. Xuan , Z. Wang , Y. Chen , et al., “Organic‐Base‐Driven Intercalation and Delamination for the Production of Functionalized Titanium Carbide Nanosheets with Superior Photothermal Therapeutic Performance,” Angewandte Chemie International Edition 55 (2016): 14569–14574. 27774723 10.1002/anie.201606643

[32] V. Kotasthane , Z. Tan , J. Yun , et al., “Selective Etching of Ti_3_AlC_2_ MAX Phases Using Quaternary Ammonium Fluorides Directly Yields Ti_3_C_2_T_z_ MXene Nanosheets: Implications for Energy Storage,” ACS Applied Nano Materials 6 (2023): 1093–1105.

[33] T. Li , L. Yao , Q. Liu , et al., “Fluorine‐Free Synthesis of High‐Purity Ti_3_C_2_T_x_ (T=OH, O) via Alkali Treatment,” Angewandte Chemie International Edition 57 (2018): 6115–6119. 29633442 10.1002/anie.201800887

[34] J. Wu , Y. Wang , Y. Zhang , et al., “Highly Safe and Ionothermal Synthesis of Ti_3_C_2_ MXene with Expanded Interlayer Spacing for Enhanced Lithium Storage,” Journal of Energy Chemistry 47 (2020): 203–209.

[35] M. Guo , W.‐C. Geng , C. Liu , J. Gu , Z. Zhang , and Y. Tang , “Ultrahigh Areal Capacitance of Flexible MXene Electrodes: Electrostatic and Steric Effects of Terminations,” Chemistry of Materials 32 (2020): 8257–8265.

[36] Y. C. Jee , J. S. Yun , S. H. Im , and W.‐S. Kim , “Environment‐friendly Synthesis of Ti_3_C_2_T_X_ MXene by Etching and Galvanic Reactions for Al Removal of Ti_3_AlC_2_ MAX,” Chemical Engineering Journal 495 (2024): 153354.

[37] S. Shah , I. Mubeen , E. Pervaiz , and H. Nasir , “Facile and Efficient Synthesis of Carboxylic Terminated Ti_3_C_2_T_x_ Nanosheets Using Citric Acid,” FlatChem 41 (2023): 100544.

[38] B. Gurzęda , N. Boulanger , A. Nordenström , C. Dejoie , and A. V. Talyzin , “Pristine MXene: in Situ XRD Study of MAX Phase Etching with HCl+ LiF Solution,” Advanced Science 11 (2024): 2408448. 39474991 10.1002/advs.202408448 PMC11672266

[39] M. Shayan Asenjan , M. Asl Farshbaf , M. K. Razavi Aghjeh , A. Tavakoli , and M. Rezaei , “Synthesis of High‐Strength TPU/MXene Nanocomposites via an in Situ Polymerization Method,” Macromolecules 57 (2024): 3993–4006.

[40] M. Han , X. Yin , H. Wu , et al., “Ti_3_C_2_ MXenes with Modified Surface for High‐Performance Electromagnetic Absorption and Shielding in the X‐Band,” ACS Applied Materials & Interfaces 8 (2016): 21011–21019. 27454148 10.1021/acsami.6b06455

[41] J.‐Y. Park , J.‐H. Lee , C.‐H. Kim , and Y.‐J. Kim , “Fabrication of Polytetrafluoroethylene Nanofibrous Membranes for Guided Bone Regeneration,” RSC Advances 8 (2018): 34359–34369. 35548619 10.1039/c8ra05637d PMC9086911

[42] I. OjaAçik , J. Madarász , M. Krunks , et al., “Thermoanalytical Studies of Titanium (IV) Acetylacetonate Xerogels with Emphasis on Evolved Gas Analysis,” Journal of Thermal Analysis and Calorimetry 88 (2007): 557–563.

[43] C. E. Shuck , M. Han , K. Maleski , et al., “Effect of Ti_3_AlC_2_ MAX Phase on Structure and Properties of Resultant Ti_3_C_2_T_x_ MXene,” ACS Applied Nano Materials 2 (2019): 3368–3376.

[44] K. M. Kang , D. W. Kim , C. E. Ren , et al., “Selective Molecular Separation on Ti_3_C_2_T_x_–Graphene Oxide Membranes during Pressure‐Driven Filtration: Comparison with Graphene Oxide and MXenes,” ACS Applied Materials & Interfaces 9 (2017): 44687–44694. 29098847 10.1021/acsami.7b10932

[45] V. Natu , M. Benchakar , C. Canaff , A. Habrioux , S. Célérier , and M. W. Barsoum , “A Critical Analysis of the X‐ray Photoelectron Spectra of Ti_3_C_2_T_z_ MXenes,” Matter 4 (2021): 1224–1251.

[46] M. Benchakar , L. Loupias , C. Garnero , et al., “One MAX Phase, Different MXenes: a Guideline to Understand the Crucial Role of Etching Conditions on Ti_3_C_2_T_x_ Surface Chemistry,” Applied Surface Science 530 (2020): 147209.

[47] Y. Zhao , G. Dong , M. Zhang , et al., “Surface‐engineered Ti_3_C_2_T_x_ MXene Enabling Rapid Sodium/Potassium Ion Storage,” 2D Materials 10 (2023): 014005.

[48] S. Oktay , Z. Kahraman , M. Urgen , and K. Kazmanli , “XPS Investigations of Tribolayers Formed on TiN and (Ti,Re)N Coatings,” Applied Surface Science 328 (2015): 255–261.

[49] Y. Chae , S. J. Kim , S.‐Y. Cho , et al., “An Investigation into the Factors Governing the Oxidation of Two‐dimensional Ti_3_C_2_ MXene,” Nanoscale 11 (2019): 8387–8393. 30984957 10.1039/c9nr00084d

[50] M. R. Lukatskaya , O. Mashtalir , C. E. Ren , et al., “Cation Intercalation and High Volumetric Capacitance of Two‐Dimensional Titanium Carbide,” Science 341 (2013): 1502–1505. 24072919 10.1126/science.1241488

[51] O. Mashtalir , M. Naguib , V. N. Mochalin , et al., “Intercalation and Delamination of Layered Carbides and Carbonitrides,” Nature Communications 4 (2013): 1716. 10.1038/ncomms2664 23591883

[52] A. Nordenström , A. Iakunkov , I. Baburin , and A. Talyzin , “Acetylation of Graphite Oxide,” Physical Chemistry Chemical Physics 22 (2020): 21059–21067. 32936159 10.1039/d0cp03573d

[53] A. A. Udovenko and N. M. Laptash , “Dynamic Orientational Disorder in Crystals of Fluoroelpasolites, Structural Refinement of (NH_4_)_3_AlF_6_, (NH_4_)_3_TiOF_5_ and Rb_2_KTiOF_5_ ,” Acta Crystallographica Section B Structural Science 67 (2011): 447–454. 22101533 10.1107/S0108768111044867

[54] O. Knop , T. S. Cameron , S. P. Deraniyagala , D. Adhikesavalu , and M. Falk , “Infrared Spectra of the Ammonium Ion in Crystals. Part XIII. Crystal Structure of (NH_4_)_2_[AlF_5_(H_2_O)] and NH_3_D^+^ Probe‐ion Spectra in (NH_4_)_2_[AlF_5_(H_2_O)], NH_4_AlF_4_, and (NH_4_)_3_ZnCl_5_, with Remarks on Structural Filiation of AMF_4_ Fluorides,” Canadian Journal of Chemistry 63 (1985): 516–525.

