# Supplementary material for: Titanium Carbide MXene Synthesis by Etching of Titanium Aluminum Carbide in Acetic Acid Solution
Source: Small. 2026 Mar 12;22(26):e14731. doi: 10.1002/smll.202514731 (PMC13155064; doi:10.1002/smll.202514731)
Supplement: Supplementary file 1 — Supporting File: smll73078‐sup‐0001‐SuppMat.docx. [file SMLL-22-e14731-s001.docx]

Supporting Information

**Titanium Carbide MXene Synthesis by Etching of Titanium Aluminum Carbide in Acetic Acid Solution**

Bartosz Gurzęda,*^[a, b]^ Nicolas Boulanger,^[a]^ Gui Li,^[a]^ , Mads Ry Vogel Jørgensen,^[c, d]^ Innokenty Kantor,^[c]^ Igor Baburin,^[e]^ Marta Petre,^[f]^ Marius Enachescu,^[f, g]^ and Alexandr V.Talyzin*^[a]^

**1. Experimental section**

**1.1. The Ti_3_AlC_2_ etching in acetic acid solutions**

The etchant for Ti_3_AlC_2_ MAX phase was prepared in 50 mL plastic centrifugation tube by dissolution of 1.52 g of ammonium fluoride (NH_4_F, ≥98.0%, Sigma-Aldrich, Germany) in 16 mL of four different mixtures of DI water and acetic acid (CH_3_COOH, glacial, ≥99%, Merck, Germany) with CH_3_COOH:H_2_O volume ratios 4:1, 3:2, 2:3, and 1:4, respectively. Additionally, the etching process in pure acetic acid was also tested. To dissolve the ammonium fluoride fully, the mixture was placed in an ultrasonic bath for 20 minutes. Then, the solution was transferred to 50 mL PTFE container, placed in a water bath, and magnetically stirred at room temperature. 0.5 g of MAX (Ti_3_AlC_2_, ≥90%, ≤40μm particle size, Merck, Germany) was firstly grinded in agate mortar for 2 minutes and then slowly added to the solution over the course of 5 min (almost no bubbling of the mixture was observed for pure acetic acid, and more intense hydrogen formation with an increase of water content was noted). The reactor was tightly covered using parafilm to reduce evaporation of etching mixture. Next, the temperature of the water bath was increased to 40 °C and the mixture was continuously stirred for 2 to 6 days, depending of the CH_3_COOH:H_2_O ratio. After etching, the solution was separated from the powder by centrifugation (4400 RPM, 20 min), and the etched material was firmly washed with DI water using a vacuum filtration setup with a PTFE membrane (0.2 μm pore size). Finally, the prepared material was dried at 60 °C on air for 2 h. The samples’ nomenclature is presented in **Table S1**.

**Table S1.** Nomenclature of the prepared samples due to the CH_3_COOH:H_2_O ratio and the time of etching.

|  | **H2O volume** | **CH3COOH volume** | **Volume ratio** | **Etching time** | **Sample name** |
| --- | --- | --- | --- | --- | --- |
| **1** | 16 mL | - | - | 6 d | MX-AcA-6d |
| **2** | 12.8 mL | 3.2 mL | 4:1 | 4 d | MX-4AcA1H2O-4d |
| **3** | 9.6 mL | 6.4 mL | 3:2 | 3 d | MX-3AcA2H2O-3d |
| **4** | 6.4 mL | 9.6 mL | 2:3 | 2 d | MX-2AcA3H2O-2d |
| **5** | 6.4 mL | 9.6 mL | 2:3 | 3 d | MX-2AcA3H2O-3d |
| **6** | 3.2 mL | 12.8 mL | 1:4 | 2 d | MX-1AcA4H2O-2d |
| **7** | 3.2 mL | 12.8 mL | 1:4 | 3 d | MX-1AcA4H2O-3d |

**1.2. In situ synchrotron XRD measurements**

In situ synchrotron radiation XRD analysis of the Ti_3_AlC_2_ etching was investigated in a specially designed 3D printed chemical reactor (**Figure S1**), facilitating the escape of gases formed during the reaction and allowing for the recording of diffraction data in transmission geometry. The PETG body of the reactor held two PET slides (50 x 24 x 0.3 mm). The distance between the PET slides was equal to 0.8 mm and was maintained by a U-shaped 3D printed TPU seal. MAX phase (~5-10 mg) was placed between two filter paper pieces (~10 x ~10 mm) and put into the reactor. The powder was held in place, gently squeezed between the PET slides, allowing the formed hydrogen gas to escape. Etching of MAX phase by NH_4_F was studied in pure acetic acid and in 60% aqueous acetic acid solution at 40 °C. Both solutions were prepared in the same way as for chemical etching in batch experiments. XRD patterns were continuously recorded after adding the etching solution to the Ti_3_AlC_2_ powder.

**1.3. Instrumentation**

Ex situ XRD characterization of materials was performed using a Panalytical X'pert X-ray diffractometer with Cu Kα radiation in Bragg-Brentano geometry. Cu Kα average (λ = 1.5418 Å) was used for the calculation of d-spacings of the low angle reflections. Fourier transform infrared (FTIR) spectra of prepared MXenes were recorded using a Bruker Vertex 80v spectrometer in ATR mode equipped with a diamond crystal under vacuum conditions. Thermogravimetric (TG) analysis was performed using a Mettler Toledo TGA/DSC1 STARe System with a heating rate of 5 °C min^−1^ under a nitrogen flow with a rate of 40 mL min^−1^. X-ray photoelectron spectroscopy (XPS) analysis were performed using a Kratos Axis Ultra electron spectrometer equipped with a delay line detector. A monochromatic 140 W Al Kα source was used as the excitation source. The binding energy scale was adjusted with respect to the C 1s line of C-Ti bond, set at 282.0 eV. In situ synchrotron radiation XRD patterns were recorded at the DanMAX beamline at the MAX IV Laboratory. The diffraction data (10 frames per 1 s) were collected using radiation wavelength of 0.61992 Å on a DECTRIS PILATUS3 X 2M CdTe area detector. The wavelength and geometry of the instrument were refined using a LaB_6_ standard (NIST SRM660c) using the program PyFAI. The raw area detector data were azimuthally integrated to intensity versus 2θ using the MatFRAIA algorithm. Raman spectra were recorded with 514 nm laser excitation using a Renishaw inVia Raman spectrometer.

**2. Supporting Figures**


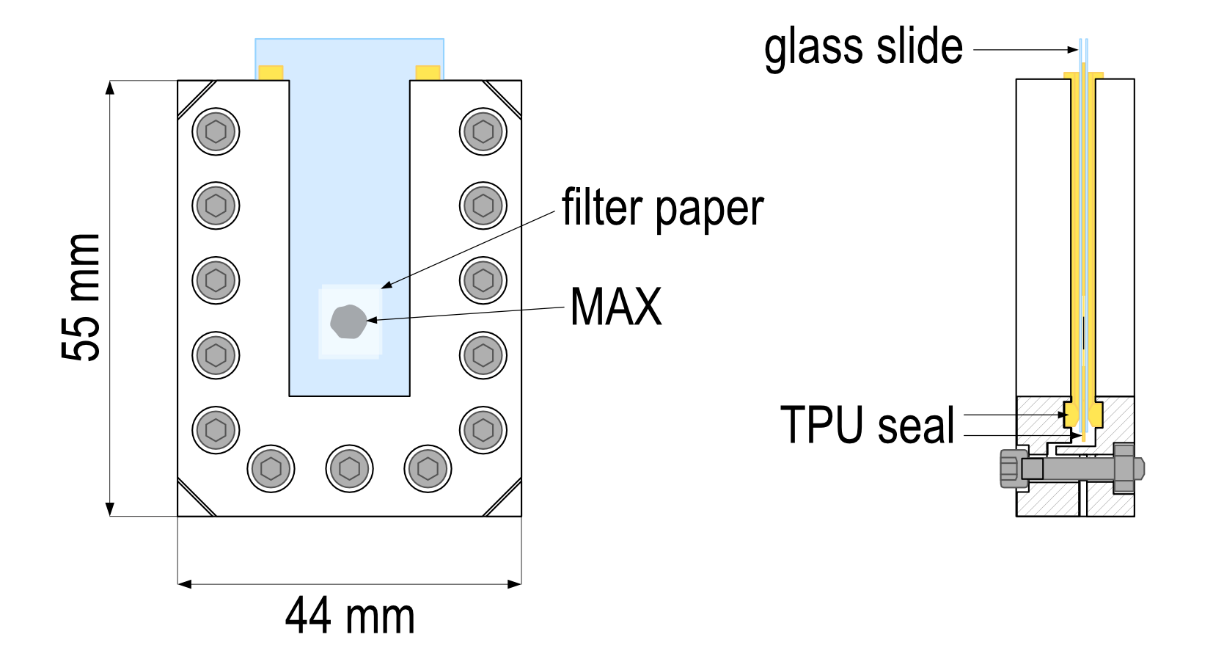


**Figure S1.** Scheme of the microscopic reactor cell designed for in situ XRD experiments.


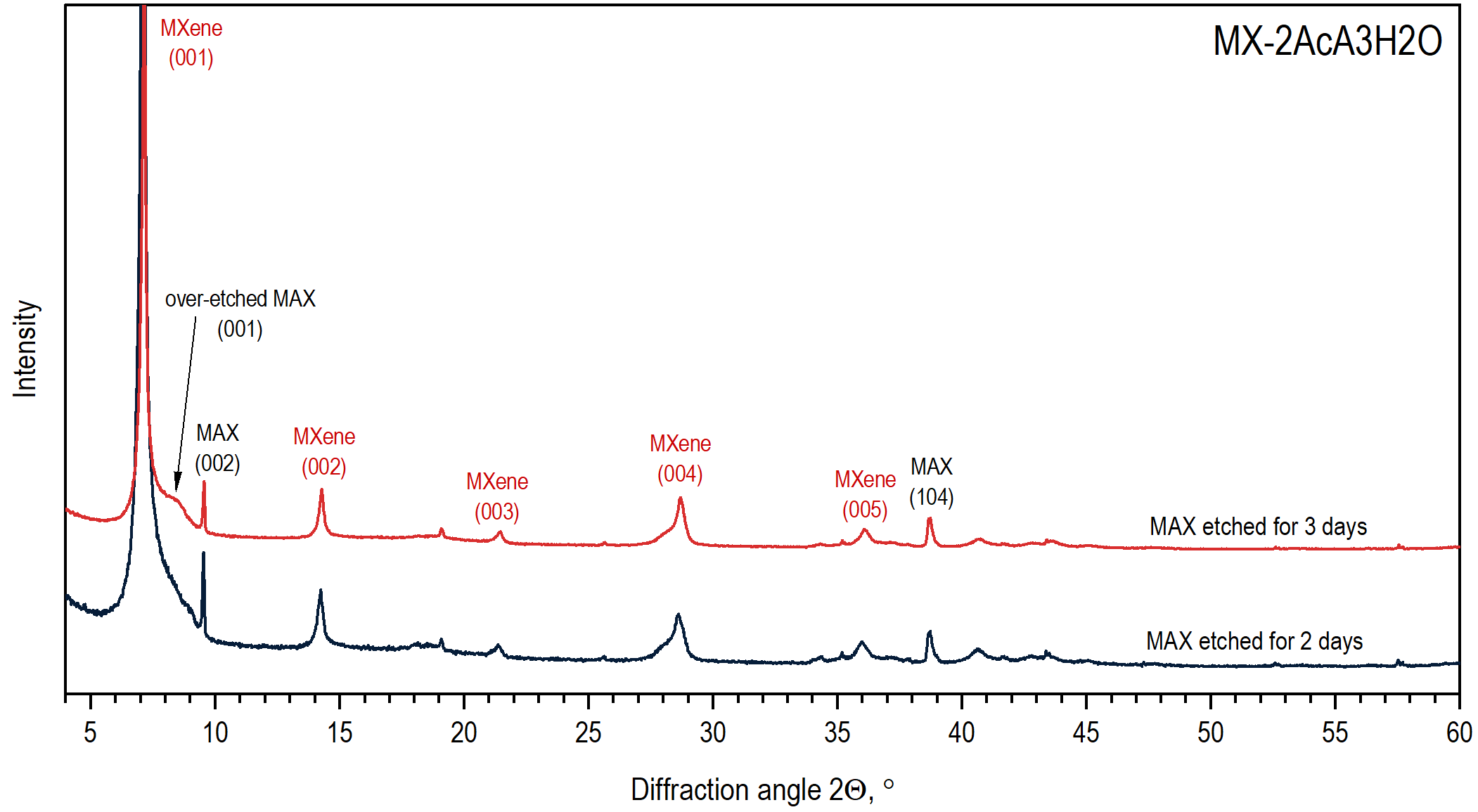


**Figure S2.** MAX phase etched by NH4F in 40% acetic acid aqueous solution by volume for
2 days (black curve) and for 3 days (red curve).


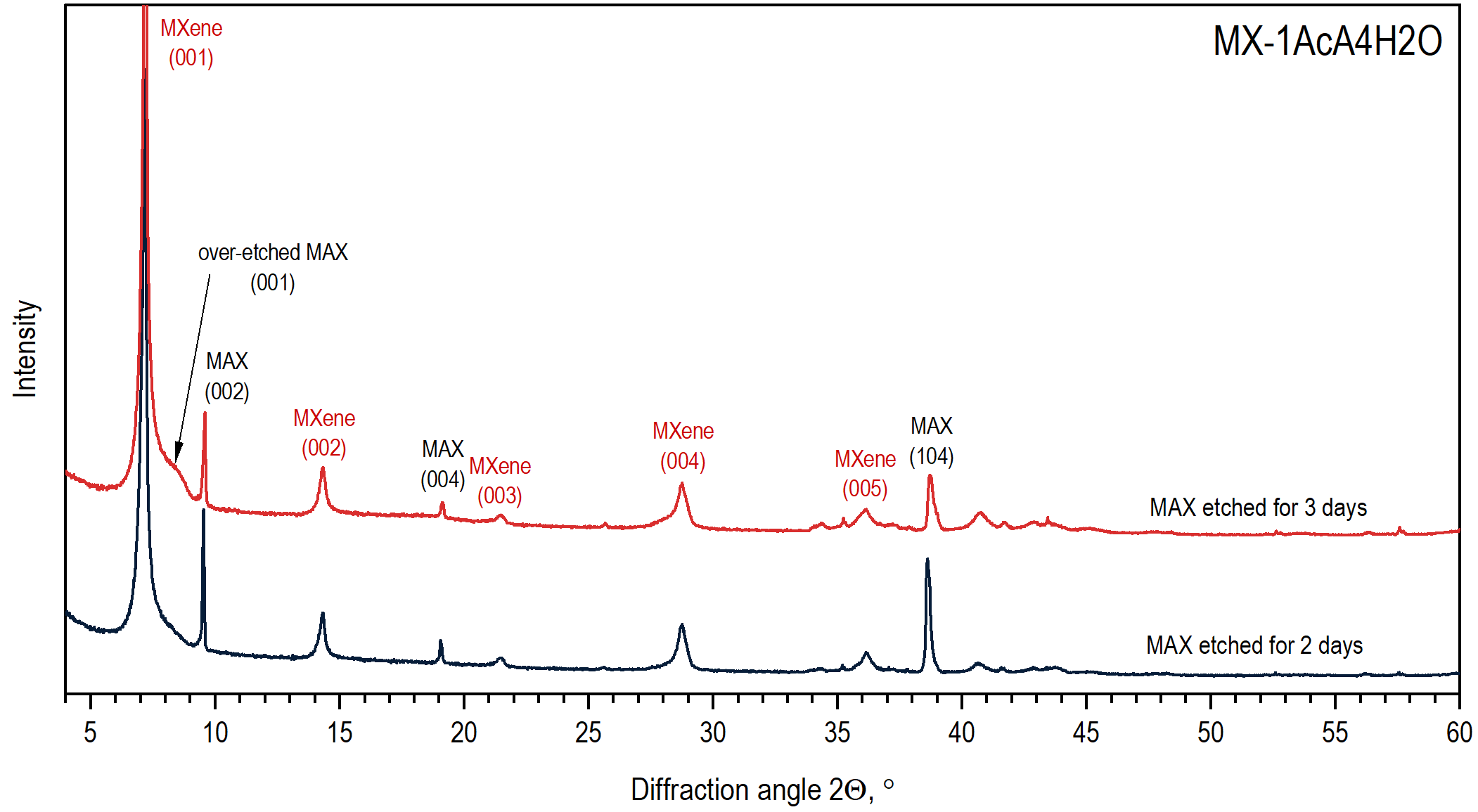


**Figure S3.** MAX phase etched by NH4F in 20% acetic acid aqueous solution by volume for
2 days (black curve) and for 3 days (red curve).


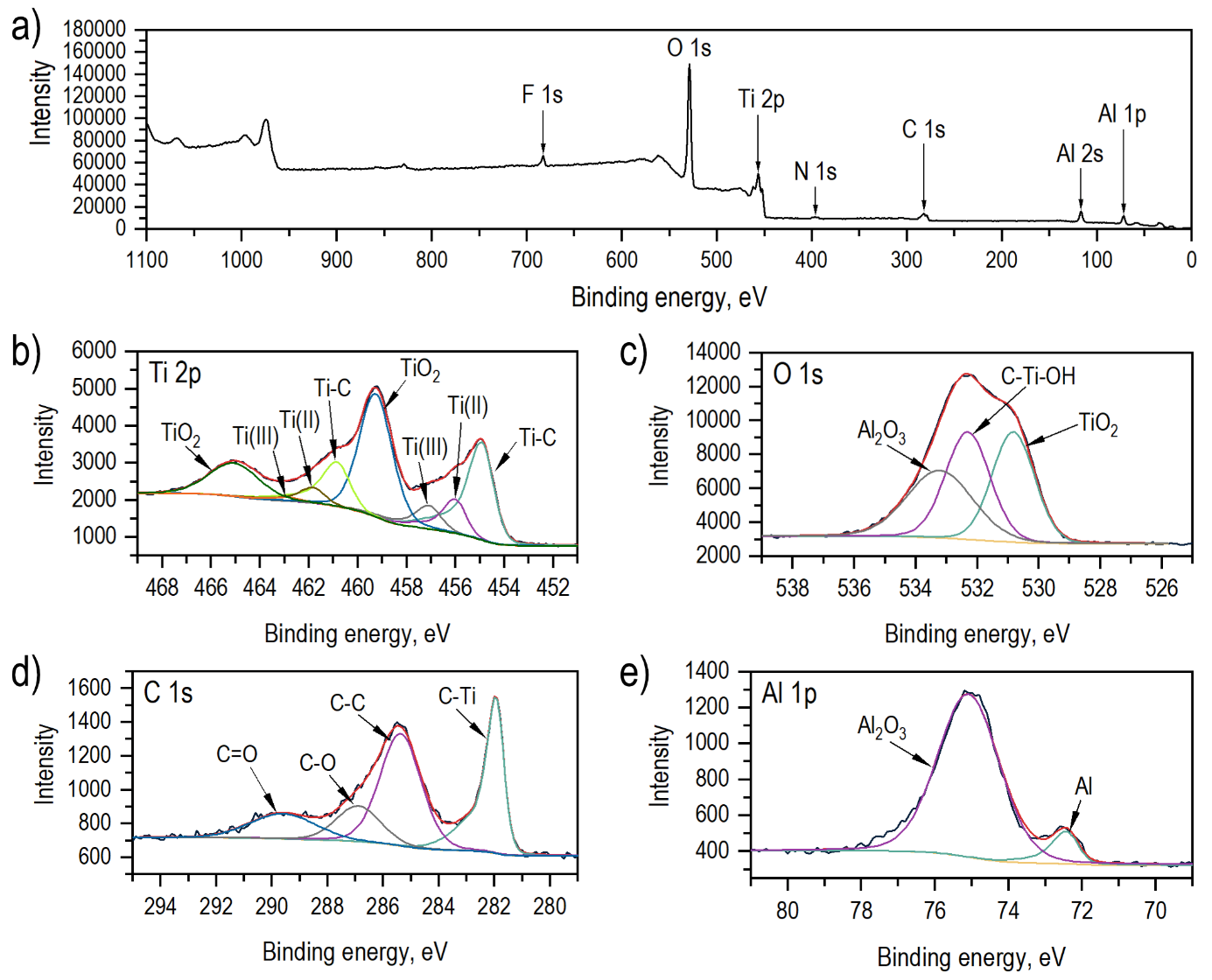


**Figure S4.** XPS analysis of Ti_3_AlC_2_ MAX phase. a) shows the survey spectra of MAX phase and b), c), d), and e) present high-resolution spectra of Ti 2p, O 1s, C 1s, and Al 1p recorded for MAX phase, respectively.^[46-47]^


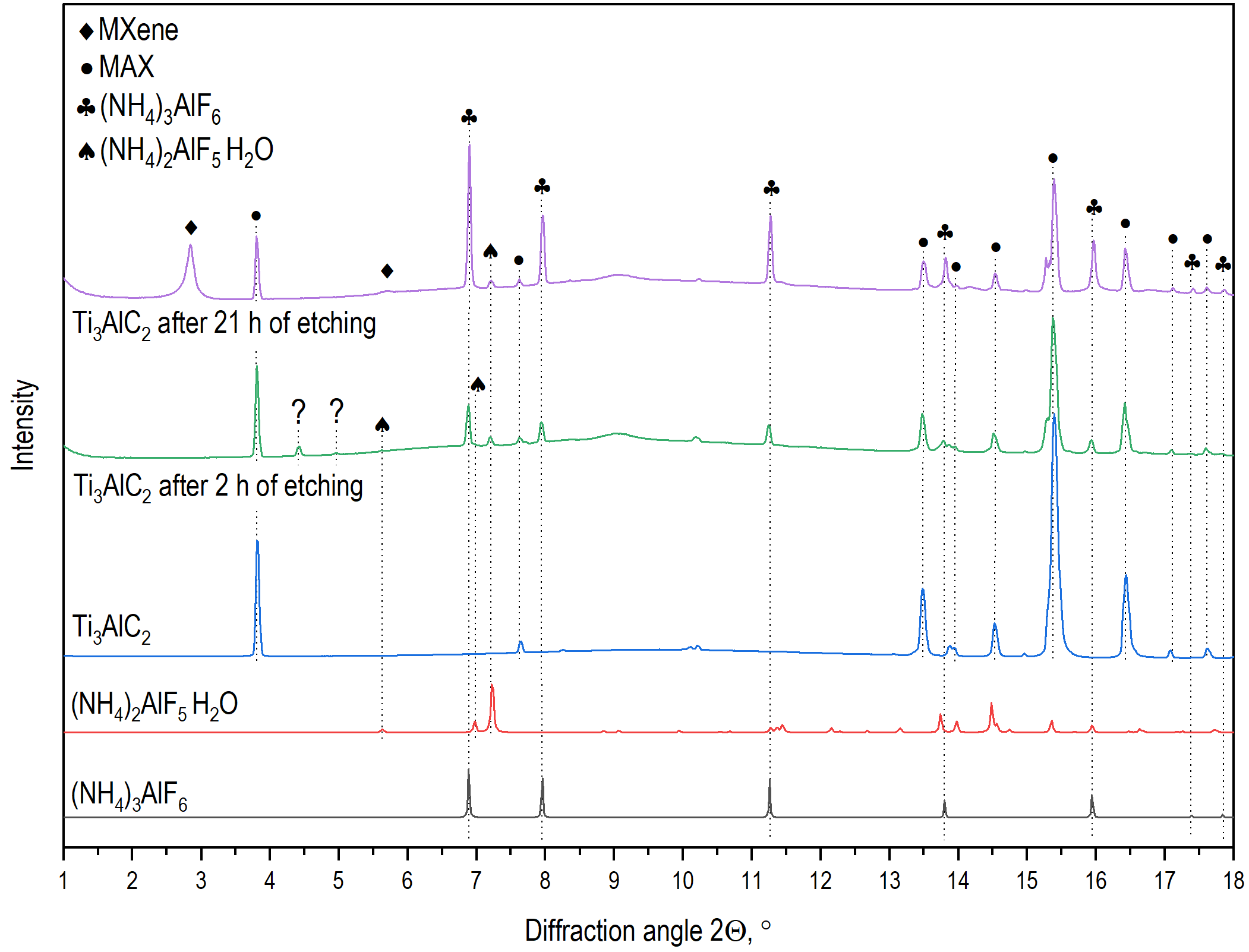


**Figure S5.** In situ recorded XRD patterns for MAX etched by NH_4_F in 60% acetic acid showing the formation of ammonium aluminum hexafluoride and ammonium aluminum pentafluoride hydrate on the surface of partially etched MAX (λ = 0.61992 Å). Black curve shows the theoretical XRD pattern simulated for (NH_4_)_3_AlF_6_ using CIF file thanks to Crystallography Open Database (COD: 2104989) and ref.^[52]^ Red curve shows the theoretical XRD pattern simulated for (NH_4_)_2_AlF_5_·H_2_O using CIF file thanks to Crystallography Open Database (COD: 1530184) and ref.^[53]^


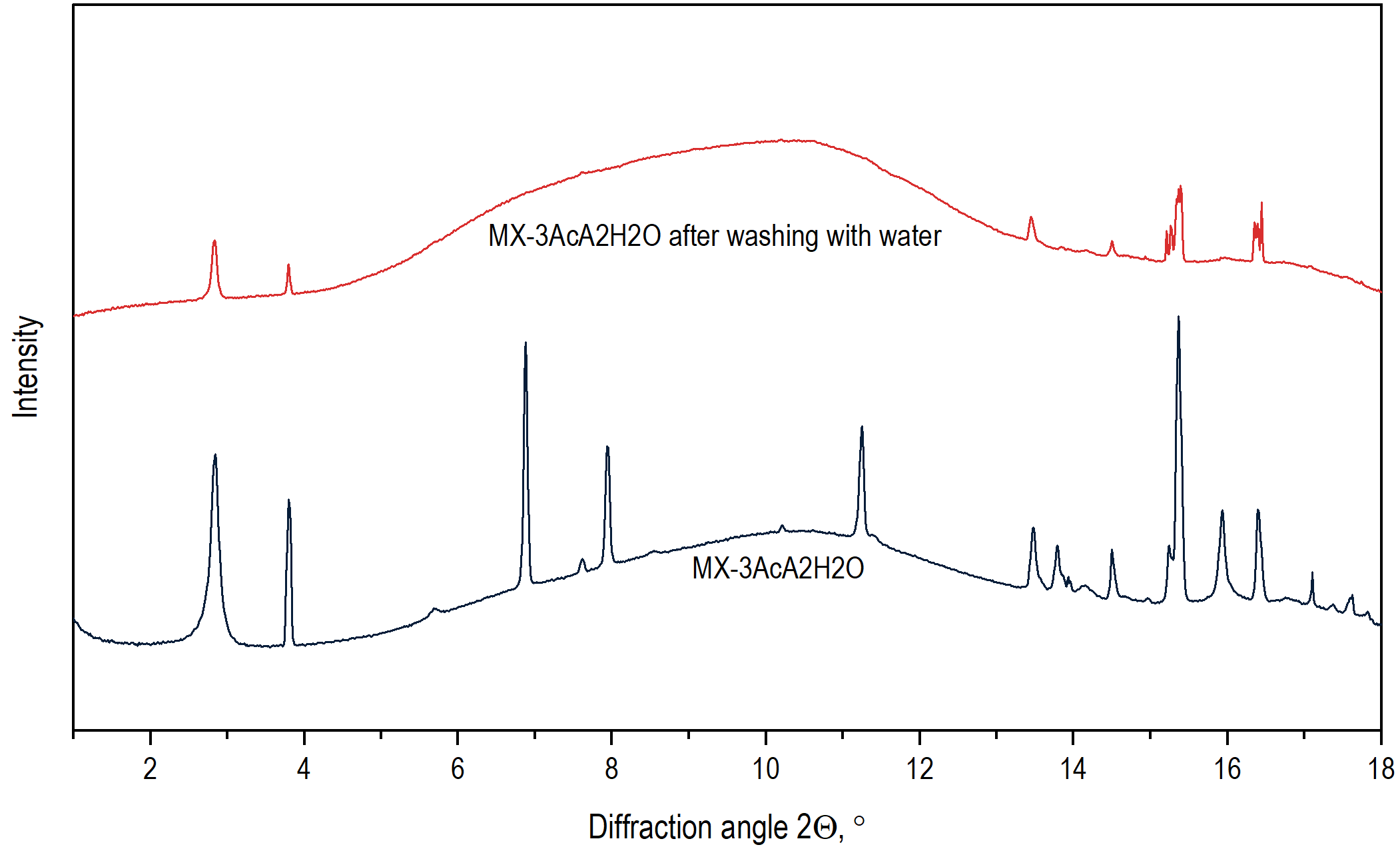


**Figure S6.** In situ recorded XRD patterns for MAX etched by NH_4_F in 60% acetic acid for
15 h (black curve) and after washing with water (red curve).

**References**

[52] A. A. Udovenko, N. M. Laptash, *Acta Crystallographica Section B* **2011**, *67*, 447-454.

[53] O. Knop, T. S. Cameron, S. P. Deraniyagala, D. Adhikesavalu, M. Falk, *Can. J. Chem.* **1985**, *63*, 516-525.
